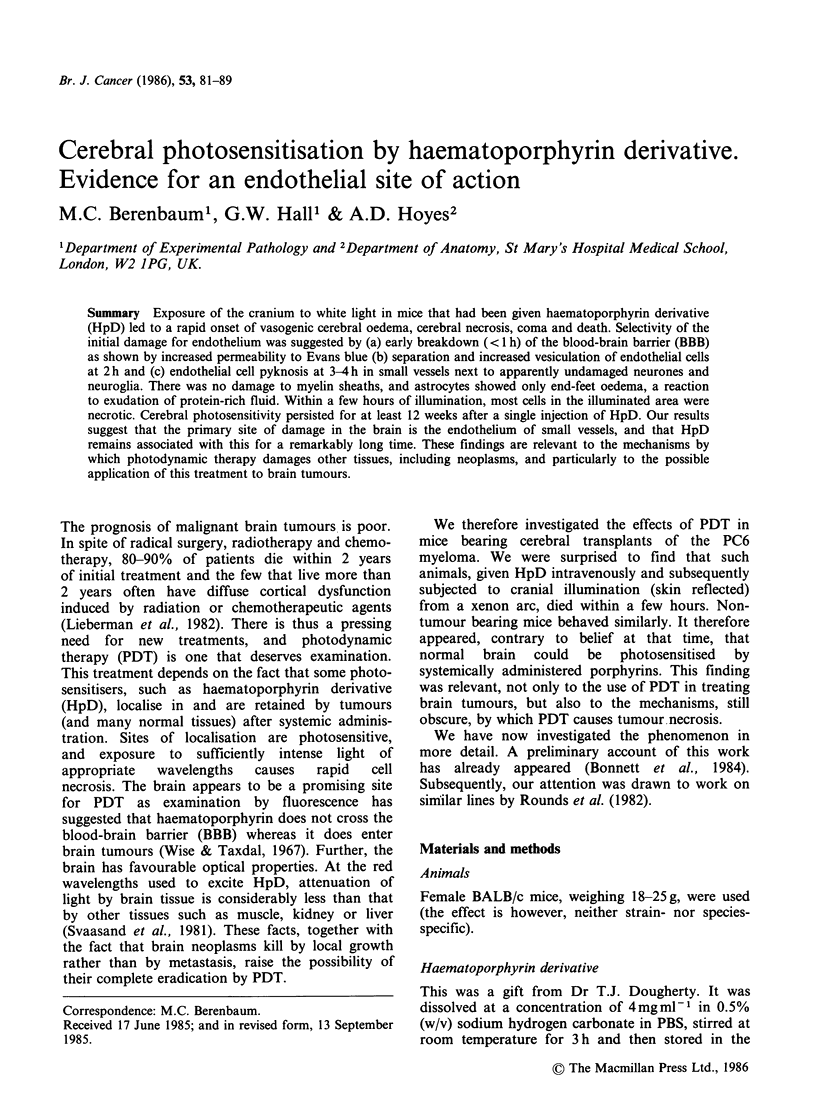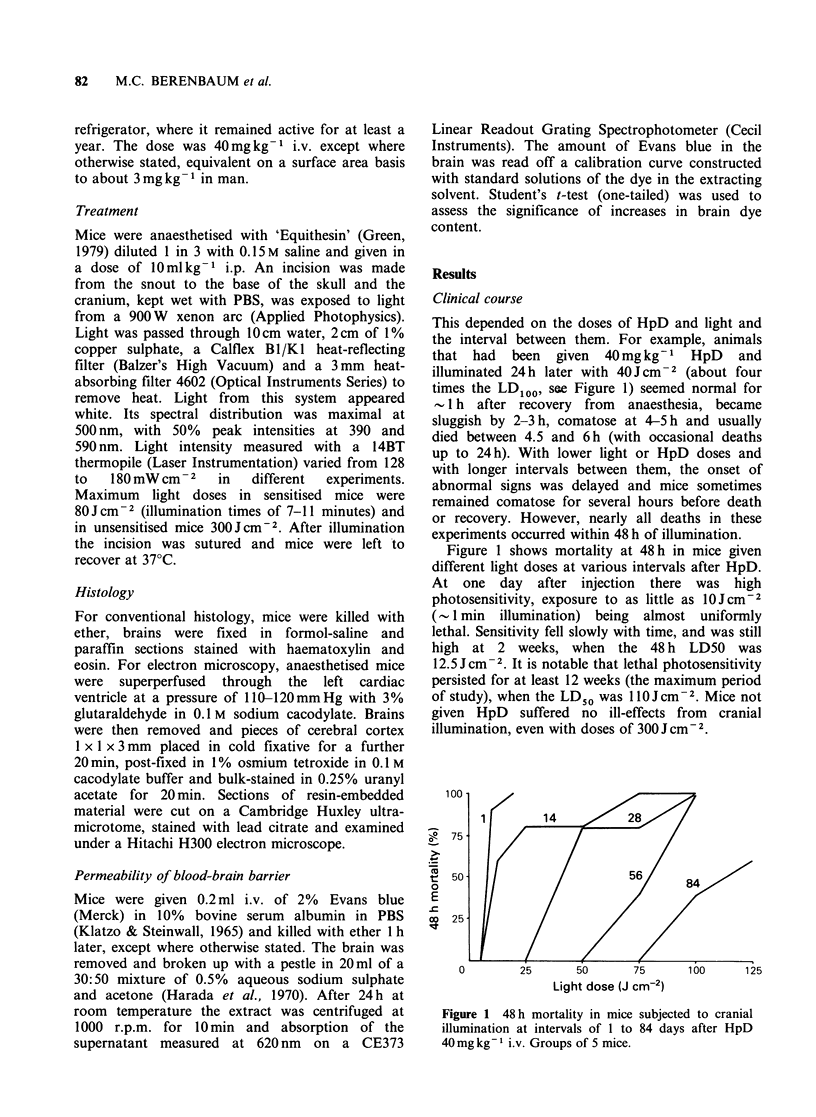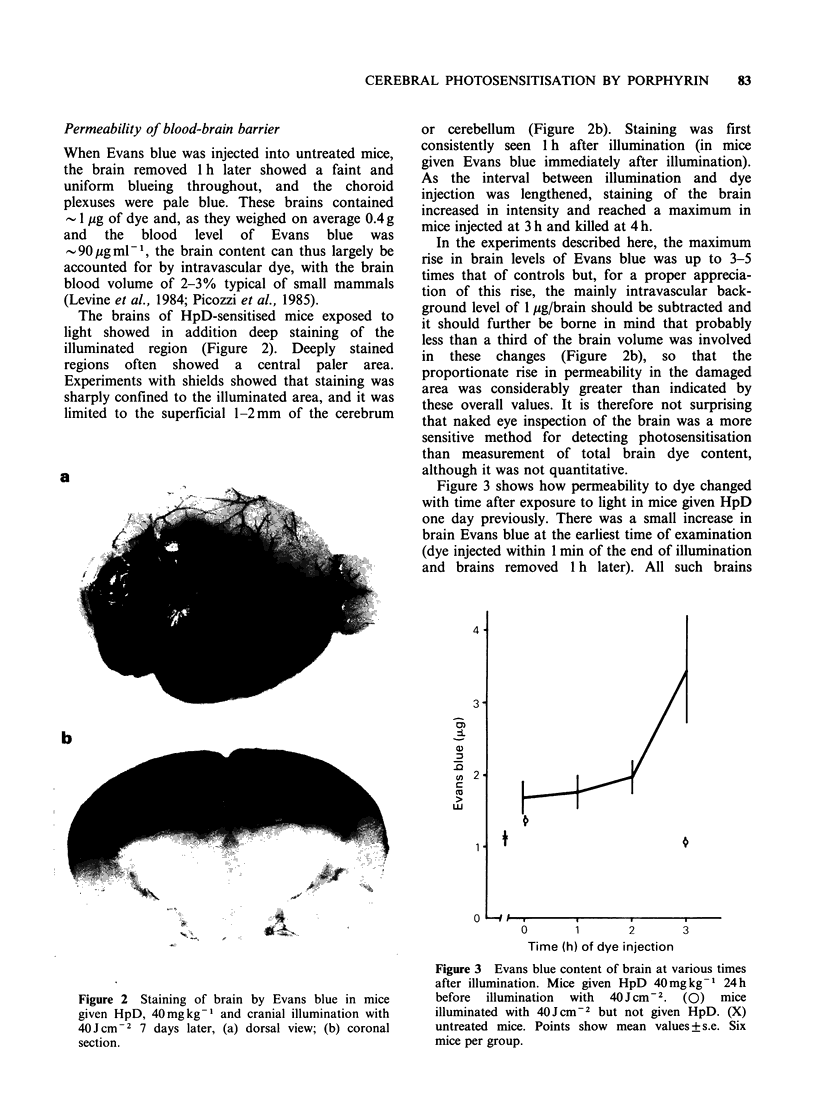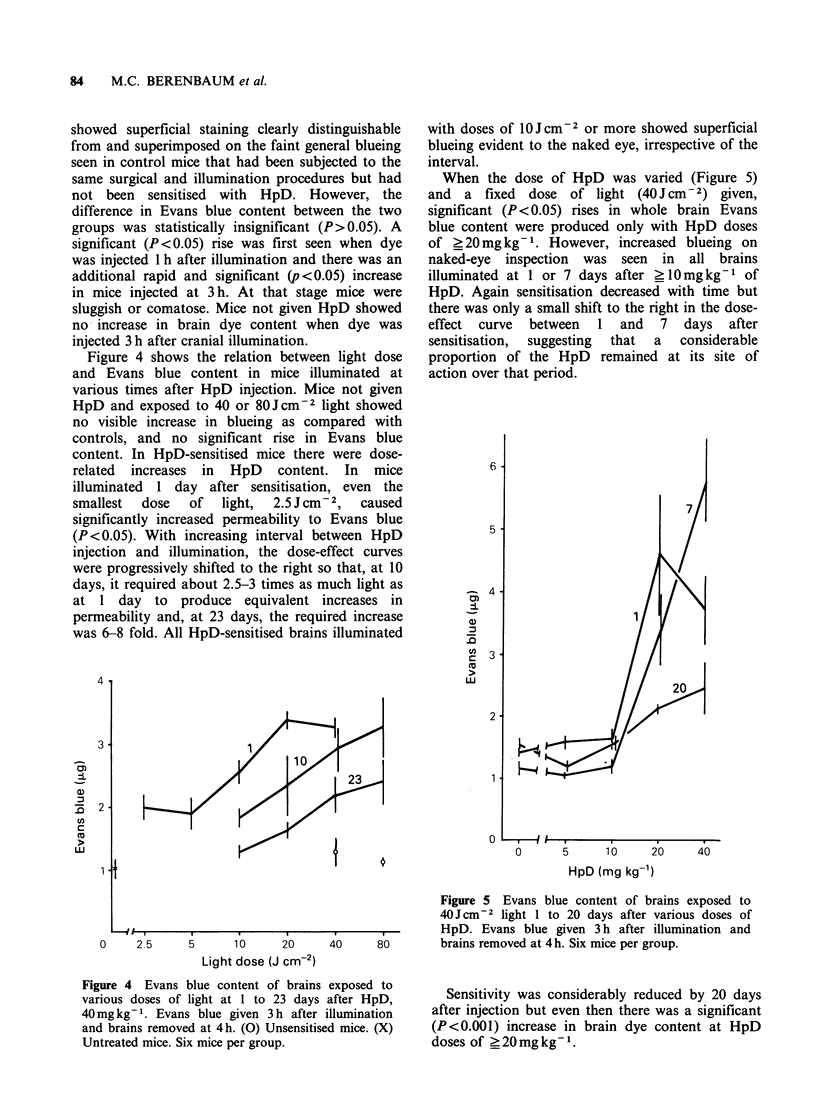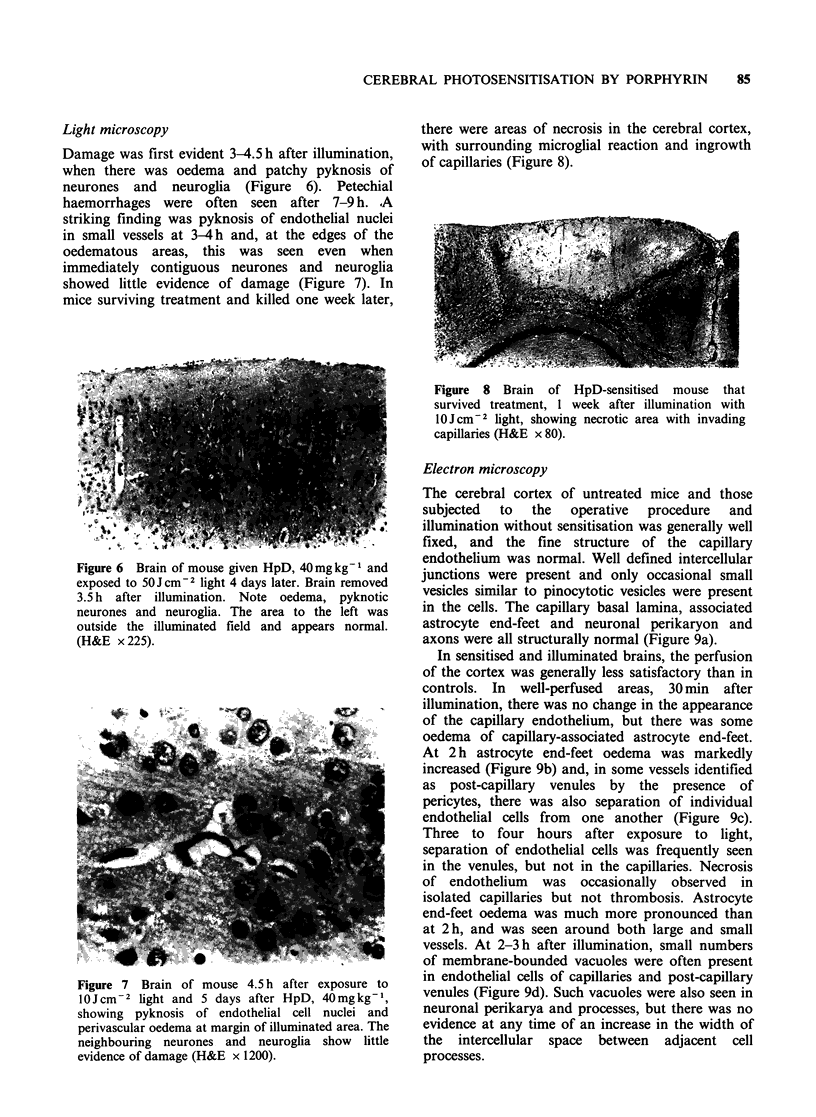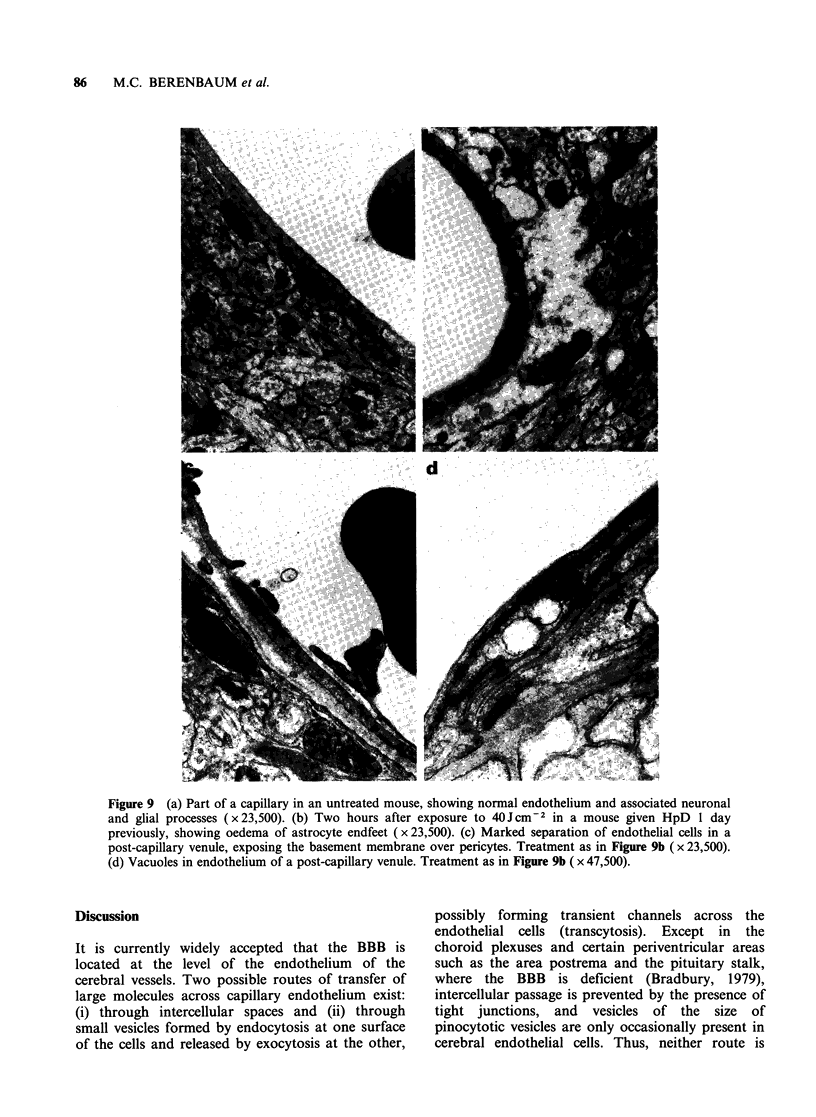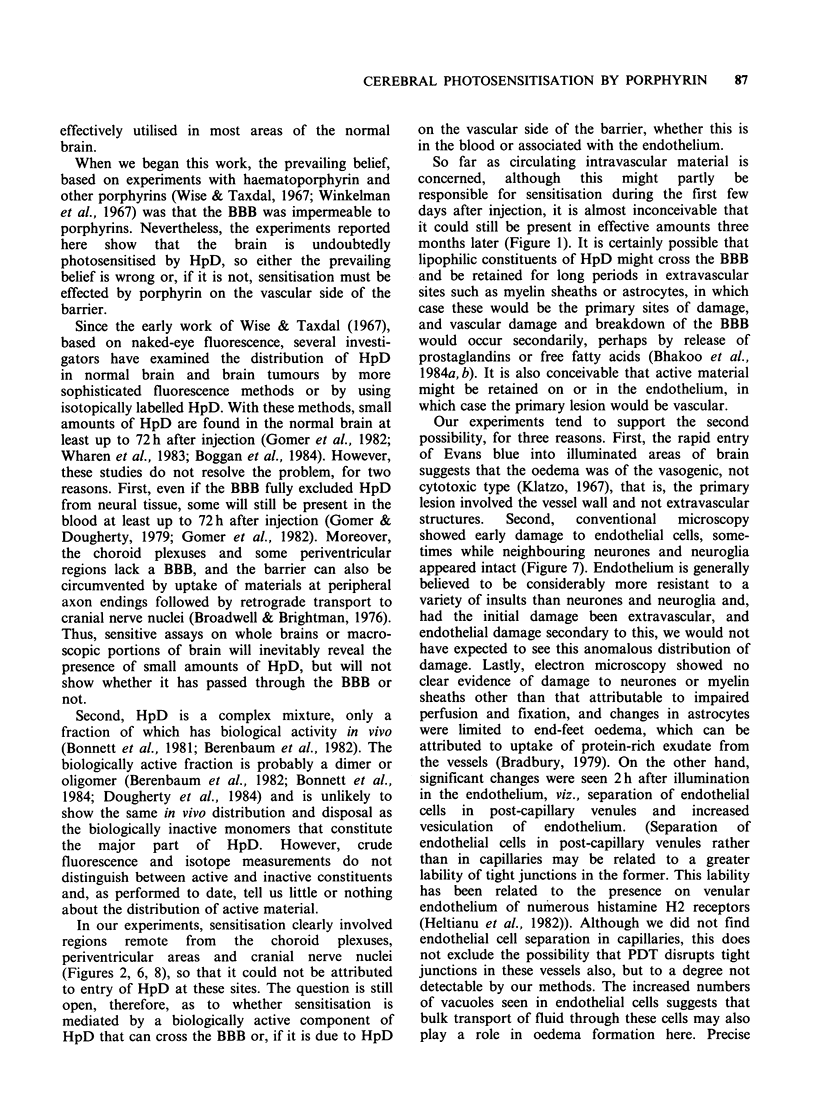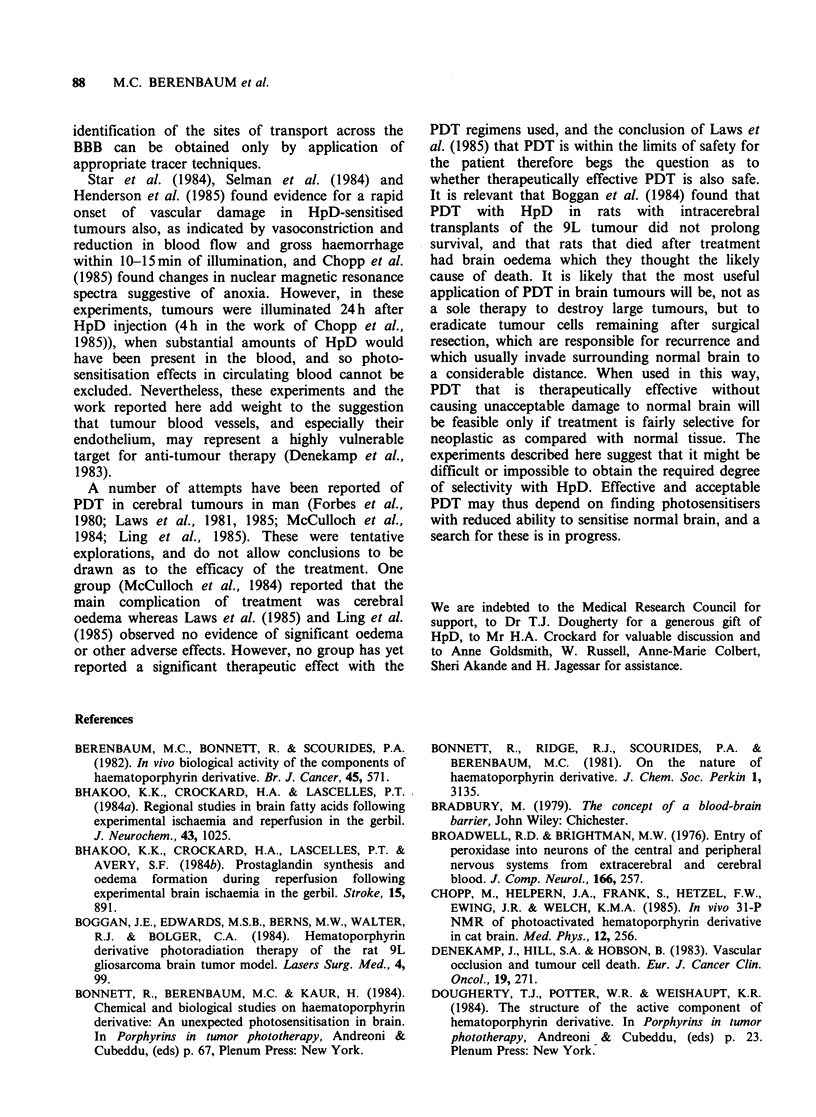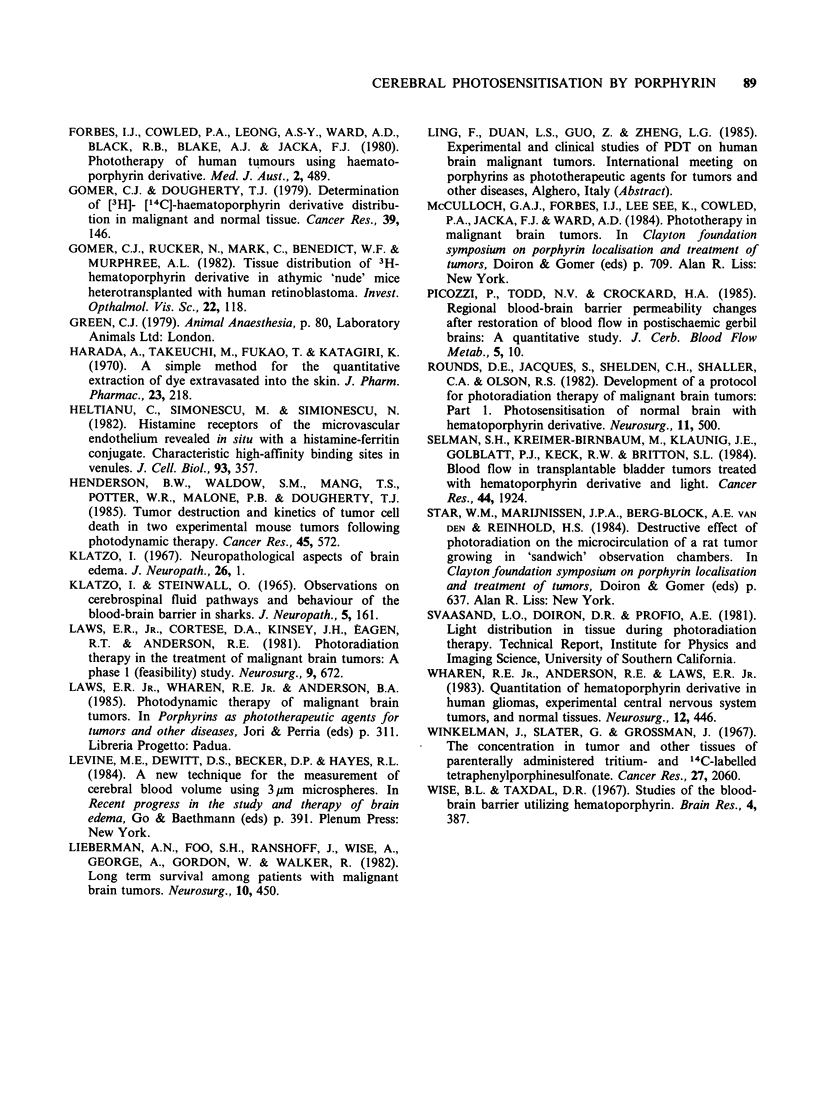# Cerebral photosensitisation by haematoporphyrin derivative. Evidence for an endothelial site of action.

**DOI:** 10.1038/bjc.1986.12

**Published:** 1986-01

**Authors:** M. C. Berenbaum, G. W. Hall, A. D. Hoyes

## Abstract

**Images:**


*Br. J. Cancer* (1986), **53**, 81–89

### Cerebral photosensitisation by haematoporphyrin derivative. Evidence for an endothelial site of action

M.C. Berenbaum[1], G.W. Hall[1] & A.D. Hoyes[2]

[1]*Department of Experimental Pathology and* [2]*Department of Anatomy, St Mary's Hospital Medical School, London, W2 1PG, UK.*

**Summary** Exposure of the cranium to white light in mice that had been given haematoporphyrin derivative (HpD) led to a rapid onset of vasogenic cerebral oedema, cerebral necrosis, coma and death. Selectivity of the initial damage for endothelium was suggested by (a) early breakdown (<1 h) of the blood-brain barrier (BBB) as shown by increased permeability to Evans blue (b) separation and increased vesiculation of endothelial cells at 2 h and (c) endothelial cell pyknosis at 3–4 h in small vessels next to apparently undamaged neurones and neuroglia. There was no damage to myelin sheaths, and astrocytes showed only end-feet oedema, a reaction to exudation of protein-rich fluid. Within a few hours of illumination, most cells in the illuminated area were necrotic. Cerebral photosensitivity persisted for at least 12 weeks after a single injection of HpD. Our results suggest that the primary site of damage in the brain is the endothelium of small vessels, and that HpD remains associated with this for a remarkably long time. These findings are relevant to the mechanisms by which photodynamic therapy damages other tissues, including neoplasms, and particularly to the possible application of this treatment to brain tumours.

The prognosis of malignant brain tumours is poor. In spite of radical surgery, radiotherapy and chemotherapy, 80–90% of patients die within 2 years of initial treatment and the few that live more than 2 years often have diffuse cortical dysfunction induced by radiation or chemotherapeutic agents (Lieberman *et al.*, 1982). There is thus a pressing need for new treatments, and photodynamic therapy (PDT) is one that deserves examination. This treatment depends on the fact that some photosensitisers, such as haematoporphyrin derivative (HpD), localise in and are retained by tumours (and many normal tissues) after systemic administration. Sites of localisation are photosensitive, and exposure to sufficiently intense light of appropriate wavelengths causes rapid cell necrosis. The brain appears to be a promising site for PDT as examination by fluorescence has suggested that haematoporphyrin does not cross the blood-brain barrier (BBB) whereas it does enter brain tumours (Wise & Taxdal, 1967). Further, the brain has favourable optical properties. At the red wavelengths used to excite HpD, attenuation of light by brain tissue is considerably less than that by other tissues such as muscle, kidney or liver (Svaasand *et al.*, 1981). These facts, together with the fact that brain neoplasms kill by local growth rather than by metastasis, raise the possibility of their complete eradication by PDT.

Correspondence: M.C. Berenbaum.
Received 17 June 1985; and in revised form, 13 September 1985.

We therefore investigated the effects of PDT in mice bearing cerebral transplants of the PC6 myeloma. We were surprised to find that such animals, given HpD intravenously and subsequently subjected to cranial illumination (skin reflected) from a xenon arc, died within a few hours. Non-tumour bearing mice behaved similarly. It therefore appeared, contrary to belief at that time, that normal brain could be photosensitised by systemically administered porphyrins. This finding was relevant, not only to the use of PDT in treating brain tumours, but also to the mechanisms, still obscure, by which PDT causes tumour necrosis.

We have now investigated the phenomenon in more detail. A preliminary account of this work has already appeared (Bonnett *et al.*, 1984). Subsequently, our attention was drawn to work on similar lines by Rounds *et al.* (1982).

### Materials and methods

### *Animals*

Female BALB/c mice, weighing 18–25 g, were used (the effect is however, neither strain- nor species-specific).

### *Haematoporphyrin derivative*

This was a gift from Dr T.J. Dougherty. It was dissolved at a concentration of $4\,mg\,ml^{-1}$ in 0.5% (w/v) sodium hydrogen carbonate in PBS, stirred at room temperature for 3 h and then stored in the

© The Macmillan Press Ltd., 1986

refrigerator, where it remained active for at least a year. The dose was 40 mg kg$^{-1}$ i.v. except where otherwise stated, equivalent on a surface area basis to about 3 mg kg$^{-1}$ in man.

*Treatment*

Mice were anaesthetised with 'Equithesin' (Green, 1979) diluted 1 in 3 with 0.15 M saline and given in a dose of 10 ml kg$^{-1}$ i.p. An incision was made from the snout to the base of the skull and the cranium, kept wet with PBS, was exposed to light from a 900 W xenon arc (Applied Photophysics). Light was passed through 10 cm water, 2 cm of 1% copper sulphate, a Calflex B1/K1 heat-reflecting filter (Balzer's High Vacuum) and a 3 mm heat-absorbing filter 4602 (Optical Instruments Series) to remove heat. Light from this system appeared white. Its spectral distribution was maximal at 500 nm, with 50% peak intensities at 390 and 590 nm. Light intensity measured with a 14BT thermopile (Laser Instrumentation) varied from 128 to 180 mW cm$^{-2}$ in different experiments. Maximum light doses in sensitised mice were 80 J cm$^{-2}$ (illumination times of 7–11 minutes) and in unsensitised mice 300 J cm$^{-2}$. After illumination the incision was sutured and mice were left to recover at 37°C.

*Histology*

For conventional histology, mice were killed with ether, brains were fixed in formol-saline and paraffin sections stained with haematoxylin and eosin. For electron microscopy, anaesthetised mice were superperfused through the left cardiac ventricle at a pressure of 110–120 mm Hg with 3% glutaraldehyde in 0.1 M sodium cacodylate. Brains were then removed and pieces of cerebral cortex 1 × 1 × 3 mm placed in cold fixative for a further 20 min, post-fixed in 1% osmium tetroxide in 0.1 M cacodylate buffer and bulk-stained in 0.25% uranyl acetate for 20 min. Sections of resin-embedded material were cut on a Cambridge Huxley ultra-microtome, stained with lead citrate and examined under a Hitachi H300 electron microscope.

*Permeability of blood-brain barrier*

Mice were given 0.2 ml i.v. of 2% Evans blue (Merck) in 10% bovine serum albumin in PBS (Klatzo & Steinwall, 1965) and killed with ether 1 h later, except where otherwise stated. The brain was removed and broken up with a pestle in 20 ml of a 30:50 mixture of 0.5% aqueous sodium sulphate and acetone (Harada *et al.*, 1970). After 24 h at room temperature the extract was centrifuged at 1000 r.p.m. for 10 min and absorption of the supernatant measured at 620 nm on a CE373 Linear Readout Grating Spectrophotometer (Cecil Instruments). The amount of Evans blue in the brain was read off a calibration curve constructed with standard solutions of the dye in the extracting solvent. Student's *t*-test (one-tailed) was used to assess the significance of increases in brain dye content.

### Results

*Clinical course*

This depended on the doses of HpD and light and the interval between them. For example, animals that had been given 40 mg kg$^{-1}$ HpD and illuminated 24 h later with 40 J cm$^{-2}$ (about four times the $LD_{100}$, see Figure 1) seemed normal for ~1 h after recovery from anaesthesia, became sluggish by 2–3 h, comatose at 4–5 h and usually died between 4.5 and 6 h (with occasional deaths up to 24 h). With lower light or HpD doses and with longer intervals between them, the onset of abnormal signs was delayed and mice sometimes remained comatose for several hours before death or recovery. However, nearly all deaths in these experiments occurred within 48 h of illumination.

Figure 1 shows mortality at 48 h in mice given different light doses at various intervals after HpD. At one day after injection there was high photosensitivity, exposure to as little as 10 J cm$^{-2}$ (~1 min illumination) being almost uniformly lethal. Sensitivity fell slowly with time, and was still high at 2 weeks, when the 48 h LD50 was 12.5 J cm$^{-2}$. It is notable that lethal photosensitivity persisted for at least 12 weeks (the maximum period of study), when the $LD_{50}$ was 110 J cm$^{-2}$. Mice not given HpD suffered no ill-effects from cranial illumination, even with doses of 300 J cm$^{-2}$.

**Figure 1** 48 h mortality in mice subjected to cranial illumination at intervals of 1 to 84 days after HpD 40 mg kg$^{-1}$ i.v. Groups of 5 mice.

*Permeability of blood-brain barrier*

When Evans blue was injected into untreated mice, the brain removed 1 h later showed a faint and uniform blueing throughout, and the choroid plexuses were pale blue. These brains contained ~1 μg of dye and, as they weighed on average 0.4 g and the blood level of Evans blue was ~90 μg ml$^{-1}$, the brain content can thus largely be accounted for by intravascular dye, with the brain blood volume of 2–3% typical of small mammals (Levine *et al.*, 1984; Picozzi *et al.*, 1985).

The brains of HpD-sensitised mice exposed to light showed in addition deep staining of the illuminated region (Figure 2). Deeply stained regions often showed a central paler area. Experiments with shields showed that staining was sharply confined to the illuminated area, and it was limited to the superficial 1–2 mm of the cerebrum or cerebellum (Figure 2b). Staining was first consistently seen 1 h after illumination (in mice given Evans blue immediately after illumination). As the interval between illumination and dye injection was lengthened, staining of the brain increased in intensity and reached a maximum in mice injected at 3 h and killed at 4 h.

**Figure 2** Staining of brain by Evans blue in mice given HpD, 40 mg kg$^{-1}$ and cranial illumination with 40 J cm$^{-2}$ 7 days later, (a) dorsal view; (b) coronal section.

In the experiments described here, the maximum rise in brain levels of Evans blue was up to 3–5 times that of controls but, for a proper appreciation of this rise, the mainly intravascular background level of 1 μg/brain should be subtracted and it should further be borne in mind that probably less than a third of the brain volume was involved in these changes (Figure 2b), so that the proportionate rise in permeability in the damaged area was considerably greater than indicated by these overall values. It is therefore not surprising that naked eye inspection of the brain was a more sensitive method for detecting photosensitisation than measurement of total brain dye content, although it was not quantitative.

Figure 3 shows how permeability to dye changed with time after exposure to light in mice given HpD one day previously. There was a small increase in brain Evans blue at the earliest time of examination (dye injected within 1 min of the end of illumination and brains removed 1 h later). All such brains

**Figure 3** Evans blue content of brain at various times after illumination. Mice given HpD 40 mg kg$^{-1}$ 24 h before illumination with 40 J cm$^{-2}$. (O) mice illuminated with 40 J cm$^{-2}$ but not given HpD. (X) untreated mice. Points show mean values ± s.e. Six mice per group.

showed superficial staining clearly distinguishable from and superimposed on the faint general blueing seen in control mice that had been subjected to the same surgical and illumination procedures but had not been sensitised with HpD. However, the difference in Evans blue content between the two groups was statistically insignificant ($P > 0.05$). A significant ($P < 0.05$) rise was first seen when dye was injected 1 h after illumination and there was an additional rapid and significant ($p < 0.05$) increase in mice injected at 3 h. At that stage mice were sluggish or comatose. Mice not given HpD showed no increase in brain dye content when dye was injected 3 h after cranial illumination.

Figure 4 shows the relation between light dose and Evans blue content in mice illuminated at various times after HpD injection. Mice not given HpD and exposed to 40 or 80 J cm$^{-2}$ light showed no visible increase in blueing as compared with controls, and no significant rise in Evans blue content. In HpD-sensitised mice there were dose-related increases in HpD content. In mice illuminated 1 day after sensitisation, even the smallest dose of light, 2.5 J cm$^{-2}$, caused significantly increased permeability to Evans blue ($P < 0.05$). With increasing interval between HpD injection and illumination, the dose-effect curves were progressively shifted to the right so that, at 10 days, it required about 2.5–3 times as much light as at 1 day to produce equivalent increases in permeability and, at 23 days, the required increase was 6–8 fold. All HpD-sensitised brains illuminated with doses of 10 J cm$^{-2}$ or more showed superficial blueing evident to the naked eye, irrespective of the interval.

When the dose of HpD was varied (Figure 5) and a fixed dose of light (40 J cm$^{-2}$) given, significant ($P < 0.05$) rises in whole brain Evans blue content were produced only with HpD doses of $\geq 20$ mg kg$^{-1}$. However, increased blueing on naked-eye inspection was seen in all brains illuminated at 1 or 7 days after $\geq 10$ mg kg$^{-1}$ of HpD. Again sensitisation decreased with time but there was only a small shift to the right in the dose-effect curve between 1 and 7 days after sensitisation, suggesting that a considerable proportion of the HpD remained at its site of action over that period.

**Figure 4** Evans blue content of brains exposed to various doses of light at 1 to 23 days after HpD, 40 mg kg$^{-1}$. Evans blue given 3 h after illumination and brains removed at 4 h. (O) Unsensitised mice. (X) Untreated mice. Six mice per group.

**Figure 5** Evans blue content of brains exposed to 40 J cm$^{-2}$ light 1 to 20 days after various doses of HpD. Evans blue given 3 h after illumination and brains removed at 4 h. Six mice per group.

Sensitivity was considerably reduced by 20 days after injection but even then there was a significant ($P < 0.001$) increase in brain dye content at HpD doses of $\geq 20$ mg kg$^{-1}$.

*Light microscopy*

Damage was first evident 3–4.5 h after illumination, when there was oedema and patchy pyknosis of neurones and neuroglia (Figure 6). Petechial haemorrhages were often seen after 7–9 h. A striking finding was pyknosis of endothelial nuclei in small vessels at 3–4 h and, at the edges of the oedematous areas, this was seen even when immediately contiguous neurones and neuroglia showed little evidence of damage (Figure 7). In mice surviving treatment and killed one week later, there were areas of necrosis in the cerebral cortex, with surrounding microglial reaction and ingrowth of capillaries (Figure 8).

**Figure 6** Brain of mouse given HpD, 40 mg kg$^{-1}$ and exposed to 50 J cm$^{-2}$ light 4 days later. Brain removed 3.5 h after illumination. Note oedema, pyknotic neurones and neuroglia. The area to the left was outside the illuminated field and appears normal. (H&E ×225).

**Figure 7** Brain of mouse 4.5 h after exposure to 10 J cm$^{-2}$ light and 5 days after HpD, 40 mg kg$^{-1}$, showing pyknosis of endothelial cell nuclei and perivascular oedema at margin of illuminated area. The neighbouring neurones and neuroglia show little evidence of damage (H&E ×1200).

**Figure 8** Brain of HpD-sensitised mouse that survived treatment, 1 week after illumination with 10 J cm$^{-2}$ light, showing necrotic area with invading capillaries (H&E ×80).

*Electron microscopy*

The cerebral cortex of untreated mice and those subjected to the operative procedure and illumination without sensitisation was generally well fixed, and the fine structure of the capillary endothelium was normal. Well defined intercellular junctions were present and only occasional small vesicles similar to pinocytotic vesicles were present in the cells. The capillary basal lamina, associated astrocyte end-feet and neuronal perikaryon and axons were all structurally normal (Figure 9a).

In sensitised and illuminated brains, the perfusion of the cortex was generally less satisfactory than in controls. In well-perfused areas, 30 min after illumination, there was no change in the appearance of the capillary endothelium, but there was some oedema of capillary-associated astrocyte end-feet. At 2 h astrocyte end-feet oedema was markedly increased (Figure 9b) and, in some vessels identified as post-capillary venules by the presence of pericytes, there was also separation of individual endothelial cells from one another (Figure 9c). Three to four hours after exposure to light, separation of endothelial cells was frequently seen in the venules, but not in the capillaries. Necrosis of endothelium was occasionally observed in isolated capillaries but not thrombosis. Astrocyte end-feet oedema was much more pronounced than at 2 h, and was seen around both large and small vessels. At 2–3 h after illumination, small numbers of membrane-bounded vacuoles were often present in endothelial cells of capillaries and post-capillary venules (Figure 9d). Such vacuoles were also seen in neuronal perikarya and processes, but there was no evidence at any time of an increase in the width of the intercellular space between adjacent cell processes.

**Figure 9** (a) Part of a capillary in an untreated mouse, showing normal endothelium and associated neuronal and glial processes ( × 23,500). (b) Two hours after exposure to 40 J $cm^{-2}$ in a mouse given HpD 1 day previously, showing oedema of astrocyte endfeet ( × 23,500). (c) Marked separation of endothelial cells in a post-capillary venule, exposing the basement membrane over pericytes. Treatment as in **Figure 9b** ( × 23,500). (d) Vacuoles in endothelium of a post-capillary venule. Treatment as in **Figure 9b** ( × 47,500).

### Discussion

It is currently widely accepted that the BBB is located at the level of the endothelium of the cerebral vessels. Two possible routes of transfer of large molecules across capillary endothelium exist: (i) through intercellular spaces and (ii) through small vesicles formed by endocytosis at one surface of the cells and released by exocytosis at the other, possibly forming transient channels across the endothelial cells (transcytosis). Except in the choroid plexuses and certain periventricular areas such as the area postrema and the pituitary stalk, where the BBB is deficient (Bradbury, 1979), intercellular passage is prevented by the presence of tight junctions, and vesicles of the size of pinocytotic vesicles are only occasionally present in cerebral endothelial cells. Thus, neither route is effectively utilised in most areas of the normal brain.

When we began this work, the prevailing belief, based on experiments with haematoporphyrin and other porphyrins (Wise & Taxdal, 1967; Winkelman *et al.*, 1967) was that the BBB was impermeable to porphyrins. Nevertheless, the experiments reported here show that the brain is undoubtedly photosensitised by HpD, so either the prevailing belief is wrong or, if it is not, sensitisation must be effected by porphyrin on the vascular side of the barrier.

Since the early work of Wise & Taxdal (1967), based on naked-eye fluorescence, several investigators have examined the distribution of HpD in normal brain and brain tumours by more sophisticated fluorescence methods or by using isotopically labelled HpD. With these methods, small amounts of HpD are found in the normal brain at least up to 72 h after injection (Gomer *et al.*, 1982; Wharen *et al.*, 1983; Boggan *et al.*, 1984). However, these studies do not resolve the problem, for two reasons. First, even if the BBB fully excluded HpD from neural tissue, some will still be present in the blood at least up to 72 h after injection (Gomer & Dougherty, 1979; Gomer *et al.*, 1982). Moreover, the choroid plexuses and some periventricular regions lack a BBB, and the barrier can also be circumvented by uptake of materials at peripheral axon endings followed by retrograde transport to cranial nerve nuclei (Broadwell & Brightman, 1976). Thus, sensitive assays on whole brains or macroscopic portions of brain will inevitably reveal the presence of small amounts of HpD, but will not show whether it has passed through the BBB or not.

Second, HpD is a complex mixture, only a fraction of which has biological activity *in vivo* (Bonnett *et al.*, 1981; Berenbaum *et al.*, 1982). The biologically active fraction is probably a dimer or oligomer (Berenbaum *et al.*, 1982; Bonnett *et al.*, 1984; Dougherty *et al.*, 1984) and is unlikely to show the same *in vivo* distribution and disposal as the biologically inactive monomers that constitute the major part of HpD. However, crude fluorescence and isotope measurements do not distinguish between active and inactive constituents and, as performed to date, tell us little or nothing about the distribution of active material.

In our experiments, sensitisation clearly involved regions remote from the choroid plexuses, periventricular areas and cranial nerve nuclei (Figures 2, 6, 8), so that it could not be attributed to entry of HpD at these sites. The question is still open, therefore, as to whether sensitisation is mediated by a biologically active component of HpD that can cross the BBB or, if it is due to HpD on the vascular side of the barrier, whether this is in the blood or associated with the endothelium.

So far as circulating intravascular material is concerned, although this might partly be responsible for sensitisation during the first few days after injection, it is almost inconceivable that it could still be present in effective amounts three months later (Figure 1). It is certainly possible that lipophilic constituents of HpD might cross the BBB and be retained for long periods in extravascular sites such as myelin sheaths or astrocytes, in which case these would be the primary sites of damage, and vascular damage and breakdown of the BBB would occur secondarily, perhaps by release of prostaglandins or free fatty acids (Bhakoo *et al.*, 1984*a*, *b*). It is also conceivable that active material might be retained on or in the endothelium, in which case the primary lesion would be vascular.

Our experiments tend to support the second possibility, for three reasons. First, the rapid entry of Evans blue into illuminated areas of brain suggests that the oedema was of the vasogenic, not cytotoxic type (Klatzo, 1967), that is, the primary lesion involved the vessel wall and not extravascular structures. Second, conventional microscopy showed early damage to endothelial cells, sometimes while neighbouring neurones and neuroglia appeared intact (Figure 7). Endothelium is generally believed to be considerably more resistant to a variety of insults than neurones and neuroglia and, had the initial damage been extravascular, and endothelial damage secondary to this, we would not have expected to see this anomalous distribution of damage. Lastly, electron microscopy showed no clear evidence of damage to neurones or myelin sheaths other than that attributable to impaired perfusion and fixation, and changes in astrocytes were limited to end-feet oedema, which can be attributed to uptake of protein-rich exudate from the vessels (Bradbury, 1979). On the other hand, significant changes were seen 2 h after illumination in the endothelium, *viz.*, separation of endothelial cells in post-capillary venules and increased vesiculation of endothelium. (Separation of endothelial cells in post-capillary venules rather than in capillaries may be related to a greater lability of tight junctions in the former. This lability has been related to the presence on venular endothelium of numerous histamine H2 receptors (Heltianu *et al.*, 1982)). Although we did not find endothelial cell separation in capillaries, this does not exclude the possibility that PDT disrupts tight junctions in these vessels also, but to a degree not detectable by our methods. The increased numbers of vacuoles seen in endothelial cells suggests that bulk transport of fluid through these cells may also play a role in oedema formation here. Precise identification of the sites of transport across the BBB can be obtained only by application of appropriate tracer techniques.

Star *et al.* (1984), Selman *et al.* (1984) and Henderson *et al.* (1985) found evidence for a rapid onset of vascular damage in HpD-sensitised tumours also, as indicated by vasoconstriction and reduction in blood flow and gross haemorrhage within 10–15 min of illumination, and Chopp *et al.* (1985) found changes in nuclear magnetic resonance spectra suggestive of anoxia. However, in these experiments, tumours were illuminated 24 h after HpD injection (4 h in the work of Chopp *et al.*, 1985)), when substantial amounts of HpD would have been present in the blood, and so photosensitisation effects in circulating blood cannot be excluded. Nevertheless, these experiments and the work reported here add weight to the suggestion that tumour blood vessels, and especially their endothelium, may represent a highly vulnerable target for anti-tumour therapy (Denekamp *et al.*, 1983).

A number of attempts have been reported of PDT in cerebral tumours in man (Forbes *et al.*, 1980; Laws *et al.*, 1981, 1985; McCulloch *et al.*, 1984; Ling *et al.*, 1985). These were tentative explorations, and do not allow conclusions to be drawn as to the efficacy of the treatment. One group (McCulloch *et al.*, 1984) reported that the main complication of treatment was cerebral oedema whereas Laws *et al.* (1985) and Ling *et al.* (1985) observed no evidence of significant oedema or other adverse effects. However, no group has yet reported a significant therapeutic effect with the PDT regimens used, and the conclusion of Laws *et al.* (1985) that PDT is within the limits of safety for the patient therefore begs the question as to whether therapeutically effective PDT is also safe. It is relevant that Boggan *et al.* (1984) found that PDT with HpD in rats with intracerebral transplants of the 9L tumour did not prolong survival, and that rats that died after treatment had brain oedema which they thought the likely cause of death. It is likely that the most useful application of PDT in brain tumours will be, not as a sole therapy to destroy large tumours, but to eradicate tumour cells remaining after surgical resection, which are responsible for recurrence and which usually invade surrounding normal brain to a considerable distance. When used in this way, PDT that is therapeutically effective without causing unacceptable damage to normal brain will be feasible only if treatment is fairly selective for neoplastic as compared with normal tissue. The experiments described here suggest that it might be difficult or impossible to obtain the required degree of selectivity with HpD. Effective and acceptable PDT may thus depend on finding photosensitisers with reduced ability to sensitise normal brain, and a search for these is in progress.

We are indebted to the Medical Research Council for support, to Dr T.J. Dougherty for a generous gift of HpD, to Mr H.A. Crockard for valuable discussion and to Anne Goldsmith, W. Russell, Anne-Marie Colbert, Sheri Akande and H. Jagessar for assistance.

### References

BERENBAUM, M.C., BONNETT, R. & SCOURIDES, P.A. (1982). *In vivo* biological activity of the components of haematoporphyrin derivative. *Br. J. Cancer*, **45**, 571.

BHAKOO, K.K., CROCKARD, H.A. & LASCELLES, P.T. (1984*a*). Regional studies in brain fatty acids following experimental ischaemia and reperfusion in the gerbil. *J. Neurochem.*, **43**, 1025.

BHAKOO, K.K., CROCKARD, H.A., LASCELLES, P.T. & AVERY, S.F. (1984*b*). Prostaglandin synthesis and oedema formation during reperfusion following experimental brain ischaemia in the gerbil. *Stroke*, **15**, 891.

BOGGAN, J.E., EDWARDS, M.S.B., BERNS, M.W., WALTER, R.J. & BOLGER, C.A. (1984). Hematoporphyrin derivative photoradiation therapy of the rat 9L gliosarcoma brain tumor model. *Lasers Surg. Med.*, **4**, 99.

BONNETT, R., BERENBAUM, M.C. & KAUR, H. (1984). Chemical and biological studies on haematoporphyrin derivative: An unexpected photosensitisation in brain. In *Porphyrins in tumor phototherapy*, Andreoni & Cubeddu, (eds) p. 67, Plenum Press: New York.

BONNETT, R., RIDGE, R.J., SCOURIDES, P.A. & BERENBAUM, M.C. (1981). On the nature of haematoporphyrin derivative. *J. Chem. Soc. Perkin* **1**, 3135.

BRADBURY, M. (1979). *The concept of a blood-brain barrier*, John Wiley: Chichester.

BROADWELL, R.D. & BRIGHTMAN, M.W. (1976). Entry of peroxidase into neurons of the central and peripheral nervous systems from extracerebral and cerebral blood. *J. Comp. Neurol.*, **166**, 257.

CHOPP, M., HELPERN, J.A., FRANK, S., HETZEL, F.W., EWING, J.R. & WELCH, K.M.A. (1985). *In vivo* 31-P NMR of photoactivated hematoporphyrin derivative in cat brain. *Med. Phys.*, **12**, 256.

DENEKAMP, J., HILL, S.A. & HOBSON, B. (1983). Vascular occlusion and tumour cell death. *Eur. J. Cancer Clin. Oncol.*, **19**, 271.

DOUGHERTY, T.J., POTTER, W.R. & WEISHAUPT, K.R. (1984). The structure of the active component of hematoporphyrin derivative. In *Porphyrins in tumor phototherapy*, Andreoni & Cubeddu, (eds) p. 23. Plenum Press: New York.

FORBES, I.J., COWLED, P.A., LEONG, A.S-Y., WARD, A.D., BLACK, R.B., BLAKE, A.J. & JACKA, F.J. (1980). Phototherapy of human tumours using haematoporphyrin derivative. *Med. J. Aust.*, **2**, 489.

GOMER, C.J. & DOUGHERTY, T.J. (1979). Determination of [$^3$H]- [$^{14}$C]-haematoporphyrin derivative distribution in malignant and normal tissue. *Cancer Res.*, **39**, 146.

GOMER, C.J., RUCKER, N., MARK, C., BENEDICT, W.F. & MURPHREE, A.L. (1982). Tissue distribution of $^3$H-hematoporphyrin derivative in athymic 'nude' mice heterotransplanted with human retinoblastoma. *Invest. Opthalmol. Vis. Sc.*, **22**, 118.

GREEN, C.J. (1979). *Animal Anaesthesia*, p. 80, Laboratory Animals Ltd: London.

HARADA, A., TAKEUCHI, M., FUKAO, T. & KATAGIRI, K. (1970). A simple method for the quantitative extraction of dye extravasated into the skin. *J. Pharm. Pharmac.*, **23**, 218.

HELTIANU, C., SIMONESCU, M. & SIMIONESCU, N. (1982). Histamine receptors of the microvascular endothelium revealed *in situ* with a histamine-ferritin conjugate. Characteristic high-affinity binding sites in venules. *J. Cell. Biol.*, **93**, 357.

HENDERSON, B.W., WALDOW, S.M., MANG, T.S., POTTER, W.R., MALONE, P.B. & DOUGHERTY, T.J. (1985). Tumor destruction and kinetics of tumor cell death in two experimental mouse tumors following photodynamic therapy. *Cancer Res.*, **45**, 572.

KLATZO, I. (1967). Neuropathological aspects of brain edema. *J. Neuropath.*, **26**, 1.

KLATZO, I. & STEINWALL, O. (1965). Observations on cerebrospinal fluid pathways and behaviour of the blood-brain barrier in sharks. *J. Neuropath.*, **5**, 161.

LAWS, E.R., JR., CORTESE, D.A., KINSEY, J.H., ÈAGEN, R.T. & ANDERSON, R.E. (1981). Photoradiation therapy in the treatment of malignant brain tumors: A phase 1 (feasibility) study. *Neurosurg.*, **9**, 672.

LAWS, E.R. JR., WHAREN, R.E. JR. & ANDERSON, B.A. (1985). Photodynamic therapy of malignant brain tumors. In *Porphyrins as phototherapeutic agents for tumors and other diseases*, Jori & Perria (eds) p. 311. Libreria Progetto: Padua.

LEVINE, M.E., DEWITT, D.S., BECKER, D.P. & HAYES, R.L. (1984). A new technique for the measurement of cerebral blood volume using 3 $\mu$m microspheres. In *Recent progress in the study and therapy of brain edema*, Go & Baethmann (eds) p. 391. Plenum Press: New York.

LIEBERMAN, A.N., FOO, S.H., RANSHOFF, J., WISE, A., GEORGE, A., GORDON, W. & WALKER, R. (1982). Long term survival among patients with malignant brain tumors. *Neurosurg.*, **10**, 450.

LING, F., DUAN, L.S., GUO, Z. & ZHENG, L.G. (1985). Experimental and clinical studies of PDT on human brain malignant tumors. International meeting on porphyrins as phototherapeutic agents for tumors and other diseases, Alghero, Italy (*Abstract*).

McCULLOCH, G.A.J., FORBES, I.J., LEE SEE, K., COWLED, P.A., JACKA, F.J. & WARD, A.D. (1984). Phototherapy in malignant brain tumors. In *Clayton foundation symposium on porphyrin localisation and treatment of tumors*, Doiron & Gomer (eds) p. 709. Alan R. Liss: New York.

PICOZZI, P., TODD, N.V. & CROCKARD, H.A. (1985). Regional blood-brain barrier permeability changes after restoration of blood flow in postischaemic gerbil brains: A quantitative study. *J. Cerb. Blood Flow Metab.*, **5**, 10.

ROUNDS, D.E., JACQUES, S., SHELDEN, C.H., SHALLER, C.A. & OLSON, R.S. (1982). Development of a protocol for photoradiation therapy of malignant brain tumors: Part 1. Photosensitisation of normal brain with hematoporphyrin derivative. *Neurosurg.*, **11**, 500.

SELMAN, S.H., KREIMER-BIRNBAUM, M., KLAUNIG, J.E., GOLBLATT, P.J., KECK, R.W. & BRITTON, S.L. (1984). Blood flow in transplantable bladder tumors treated with hematoporphyrin derivative and light. *Cancer Res.*, **44**, 1924.

STAR, W.M., MARIJNISSEN, J.P.A., BERG-BLOCK, A.E. VAN DEN & REINHOLD, H.S. (1984). Destructive effect of photoradiation on the microcirculation of a rat tumor growing in 'sandwich' observation chambers. In *Clayton foundation symposium on porphyrin localisation and treatment of tumors*, Doiron & Gomer (eds) p. 637. Alan R. Liss: New York.

SVAASAND, L.O., DOIRON, D.R. & PROFIO, A.E. (1981). Light distribution in tissue during photoradiation therapy. Technical Report, Institute for Physics and Imaging Science, University of Southern California.

WHAREN, R.E. JR., ANDERSON, R.E. & LAWS, E.R. JR. (1983). Quantitation of hematoporphyrin derivative in human gliomas, experimental central nervous system tumors, and normal tissues. *Neurosurg.*, **12**, 446.

WINKELMAN, J., SLATER, G. & GROSSMAN, J. (1967). The concentration in tumor and other tissues of parenterally administered tritium- and $^{14}$C-labelled tetraphenylporphinesulfonate. *Cancer Res.*, **27**, 2060.

WISE, B.L. & TAXDAL, D.R. (1967). Studies of the blood-brain barrier utilizing hematoporphyrin. *Brain Res.*, **4**, 387.